# Fasting as Cancer Treatment: Myth or Breakthrough in Oncology

**DOI:** 10.7759/cureus.81395

**Published:** 2025-03-29

**Authors:** Ghizal Fatima, Abbas A Mehdi, Jan Fedacko, Najah Hadi, Aminat Magomedova, Ammar Mehdi

**Affiliations:** 1 Department of Public Health, Era's Lucknow Medical College and Hospital, Era University, Lucknow, IND; 2 Department of Biochemistry, Era's Lucknow Medical College and Hospital, Era University, Lucknow, IND; 3 Department of Cardiology, Pavol Jozef Safarik University, Kosice, Kosice, SVK; 4 Department of Medicine, University of Kufa, Najaf, IRQ; 5 Department of Population, Moscow State University, Moscow, RUS; 6 Department of Pediatric Dentistry, Career Post Graduate Institute of Dental Sciences and Hospital, Lucknow, IND

**Keywords:** autophagy, cancer treatment, chemotherapy, fasting, metabolism

## Abstract

The concept of fasting as a potential cancer treatment has garnered increasing interest, particularly in light of emerging evidence linking dietary interventions to cancer progression and therapy outcomes. This article explores whether fasting, either intermittent or prolonged, can be a viable standalone treatment for cancer or if its therapeutic potential lies in its adjunctive role. Current research suggests that fasting induces a metabolic shift, which may inhibit cancer cell proliferation by depriving them of essential nutrients. Additionally, fasting has been shown to enhance the body's stress resistance, promote autophagy, and possibly make cancer cells more vulnerable to standard treatments such as chemotherapy and radiotherapy. However, the application of fasting as a sole treatment for cancer remains controversial and lacks substantial clinical validation. While animal models and in vitro studies indicate promising results, the translation to human trials is complex, with various types of cancer responding differently to dietary interventions. Moreover, concerns about malnutrition, loss of muscle mass, and the overall health of cancer patients undergoing fasting without supervision must be addressed. The paper critically examines the myth and reality surrounding fasting as a cancer treatment, reviewing key studies and clinical trials to provide a comprehensive understanding of its efficacy and safety. While fasting may hold promise as a supportive therapy, particularly in combination with traditional treatments, there is currently insufficient evidence to support its use as a primary treatment modality. Further research is needed to establish the parameters in which fasting might be beneficial, such as specific cancer types, patient populations, and optimal fasting regimens. Thus, while the idea of fasting as a cancer breakthrough is compelling, it remains a complementary approach rather than a standalone solution in oncology.

## Introduction and background

Cancer is one of the most dreadful diseases in the world, causing a whooping increase in morbidity and mortality worldwide regardless of intense human development. It is characterized by the uncontrolled growth of aberrant cells, afflicting 19.3 million people and causing almost 10 million people deaths globally in 2024 [1]. The concept of fasting as a cancer treatment has sparked significant debate and intrigue in both the medical community and the general public. Fasting, an ancient practice often associated with religious or cultural traditions, has gained renewed attention in recent years due to its potential therapeutic benefits in modern medicine, particularly oncology. The underlying hypothesis is that fasting alters the body's metabolic state, creating conditions that may inhibit cancer growth and enhance the efficacy of conventional treatments [2]. Proponents of fasting argue that it can induce autophagy, a process where the body clears damaged cells and regenerates healthier ones, and that it may make cancer cells more vulnerable to treatment by depriving them of glucose and other essential nutrients needed for rapid proliferation [2]. In addition, fasting is believed to trigger a stress-resistant state in normal cells, offering protection against the toxic side effects of chemotherapy and radiotherapy, while selectively targeting cancer cells, which are less adaptable to nutrient scarcity [3]. These concepts are grounded in research that shows how cancer cells exhibit altered metabolism, often referred to as the "Warburg effect," where they preferentially rely on glycolysis for energy even in the presence of oxygen. By restricting glucose availability through fasting, it is thought that the growth of cancer cells may be slowed or halted [4].

Despite the intriguing scientific basis, the idea of using fasting as a primary treatment for cancer remains controversial. Much of the current evidence supporting fasting’s anti-cancer effects comes from animal studies and in vitro experiments, with limited large-scale human trials to corroborate these findings. In rodent models, fasting has shown promise in reducing tumor growth, improving the effectiveness of chemotherapy, and enhancing overall survival [5]. However, translating these results to humans is complex due to differences in physiology, tumor types, and individual patient health statuses. While some small clinical trials have suggested that short-term fasting or fasting-mimicking diets may improve patients' tolerance to chemotherapy and reduce side effects, the long-term safety and efficacy of such interventions in treating cancer remain uncertain [6]. Furthermore, the type of fasting regimen whether intermittent fasting, prolonged fasting, or calorie restriction adds another layer of complexity to the discussion, as different cancers and patients may respond differently to various fasting protocols.

Another concern with fasting as a cancer treatment is its potential for malnutrition, particularly in cancer patients who are already at risk of weight loss and muscle wasting due to their disease. Cancer cachexia, a syndrome characterized by severe muscle loss and fat depletion, affects many cancer patients and can significantly impact treatment outcomes and quality of life [7]. Fasting, if not carefully managed, could exacerbate these issues, leading to weakened immune function, reduced tolerance to treatment, and overall poorer prognosis. For these reasons, many oncologists are cautious about recommending fasting as a therapeutic strategy, particularly outside of controlled clinical settings. While the idea of fasting as a cancer treatment is compelling and has generated considerable interest, it remains largely experimental at this stage [7]. The potential benefits of fasting, especially as an adjunct to conventional therapies, are worthy of further exploration, but there is insufficient evidence to support its use as a standalone treatment. More robust clinical trials are needed to determine the efficacy, safety, and optimal conditions under which fasting might be beneficial in cancer care. In this review, we will explore the myths and breakthrough of fasting in cancer treatment.

## Review

Mechanistic insights: fasting affects cancer cells

Fasting has been studied as a potential therapeutic approach for cancer due to its profound effects on cellular metabolism in cancer and the rest of the diseases, which may inhibit cancer cell growth and enhance the effectiveness of traditional treatments. The mechanisms behind fasting suggest that it induces a metabolic shift, altering both the behavior of cancer cells and the body’s overall response to cancer therapy. Autophagy not only helps in cleaning dysfunctional cells but also promotes cellular repair and regeneration, contributing to a healthier cellular environment in the body (Table 1).

**Table 1:** Intricate details on fasting's role in oncology, its mechanisms, limitations, and future potential, aligning with advanced scientific discourse. Sources: Refs

Aspect	Details	Scientific Implications
Potential Benefits		
Enhances Chemotherapy	Fasting induces a stress-resistant state in normal cells, reducing chemotherapy toxicity, while cancer cells become more vulnerable.	Improves differential stress resistance, allowing more effective targeting of cancer cells during treatment [11].
Reduces Side Effects	Studies show reduced fatigue, nausea, and gastrointestinal discomfort in patients undergoing short-term fasting during chemotherapy.	Enhances patient tolerance to treatment, potentially allowing higher or more frequent treatment doses [3].
Induces Autophagy	Fasting triggers cellular recycling mechanisms that clear damaged organelles and proteins, including dysfunctional mitochondria in cancer cells.	Promotes cell homeostasis and may inhibit tumor growth by eliminating damaged cells before they proliferate [8,9].
Modifies Tumor Metabolism	Cancer cells depend on glucose for survival (Warburg effect). Fasting deprives them of glucose, disrupting their metabolic pathways.	Glucose deprivation reduces cancer cell proliferation while normal cells adapt to alternative energy sources [8].
Boosts Immune Response	Rodent studies show increased activity of natural killer (NK) cells and cytotoxic T cells during fasting.	Enhanced immune surveillance improves the body’s natural ability to identify and destroy cancer cells [21,22].
Challenges and Limitations		
Malnutrition Risk	Cancer patients often experience cachexia or severe weight loss; fasting may exacerbate this condition.	Increases the risk of complications such as muscle wasting and weakened immune function [23].
Limited Human Data	Most evidence comes from preclinical animal studies and small-scale human trials.	Insufficient large-scale clinical trials to establish fasting as a standardized therapy [30].
Not a Standalone Therapy	Fasting alone is unlikely to induce tumor remission; it may only complement conventional treatments like chemotherapy and radiation.	Requires integration with existing treatment protocols and ongoing medical supervision [30].
Adherence Challenges	Patients may struggle to maintain fasting regimens due to hunger, fatigue, or existing health conditions.	Practicality issues limit its widespread implementation in clinical oncology [31].
Potential Adverse Effects	Extended fasting could lead to electrolyte imbalances, hypoglycemia, and weakened immunity.	Increased risk of complications, especially in already fragile cancer patients [23].
Future Directions		
Clinical Trials	Larger, randomized controlled trials are needed to validate fasting’s safety and efficacy as a complementary therapy.	This could provide robust evidence to support the use of fasting in oncology [31].
Personalized Approaches	Fasting regimens may need to be tailored to individual patient profiles, including cancer type, stage, and overall health.	Precision oncology could integrate fasting as part of a multidisciplinary approach to treatment [33].
Combination Strategies	Exploring fasting in combination with immunotherapies, targeted therapies, or fasting-mimicking diets.	Synergistic effects could amplify treatment outcomes while minimizing side effects [34].

Role of autophagy

Autophagy is crucial in maintaining cellular homeostasis by breaking down and recycling damaged organelles, proteins, and other cellular debris. In fasting, autophagy is unregulated as the body shifts its focus from an anabolic, the building state of the body, to a catabolic, the breakdown state of the body, utilizing the internal resources for energy. This self-digestive process (autophagy) is particularly important in clearing cancerous cells that are more susceptible to damage and dysfunction when compared to normal cells. Cancer cells, typically having higher rates of growth and proliferation, accumulate a significant amount of metabolic waste and damaged cellular components. Further, by enhancing autophagy through fasting, the body may effectively clear out all the damaged cells, reducing tumor growth [8].

Fasting triggers a unique stress response in normal cells, making them more resistant to the harmful effects of radiotherapy and chemotherapy. While cancerous cells are highly sensitive to nutrient availability, normal cells can enter a protective state during fasting, reducing their metabolic activity and enhancing their ability to withstand environmental stressors. Moreover, this selective protection of normal cells is one of the key benefits of fasting, as it could potentially reduce the side effects of cancer treatments while allowing the treatments to more effectively target cancer cells [8,9].

The Warburg effect: impact on cancer cell metabolism

The Warburg effect is yet another important mechanism through which fasting may impact cancer cells by targeting their altered metabolic pathways. Cancer cells exhibit a phenomenon commonly known as the Warburg effect, where they truly rely on glycolysis for energy production, even in the presence of oxygen [10]. This metabolic reprogramming permits cancer cells to generate energy rapidly, which is important to sustain their rapid growth and proliferation. However, this dependence on glycolysis also makes cancer cells more vulnerable to changes in nutrient availability, particularly in the availability of glucose. In fasting, the body’s glucose levels decrease, directly forcing cells to rely on alternative sources of energy, such as fatty acids and ketone bodies. Normal cells can efficiently switch to these alternative metabolic pathways, but cancer cells, due to their reliance on glycolysis, struggle to adapt [10]. This metabolic vulnerability creates an opportunity for fasting to suppress cancer cell growth by depriving them of the glucose they need for survival and proliferation. Studies have shown that fasting can reduce glucose and insulin-like growth factor 1 (IGF-1) levels, both of which are critical for cancer cell metabolism and growth. Lower levels of IGF-1, in particular, have been associated with reduced tumor growth and improved responses to chemotherapy [10].

Enhancing chemotherapy and radiotherapy

Fasting also enhances the effectiveness of conventional cancer treatments such as chemotherapy and radiotherapy by sensitizing cancer cells to these therapies. As fasting induces metabolic stress in cancer cells, it compromises their ability to repair DNA damage, making them more susceptible to the effects of chemotherapy and radiation. Radiotherapy and chemotherapy work by inducing DNA damage in rapidly dividing cells, and cancer cells, with their already impaired metabolic and repair mechanisms, are less able to recover from such damage during periods of fasting [11]. Furthermore, fasting induces a shift in the immune system that may further promote anti-cancer activity. In fasting, the production of pro-inflammatory cytokines decreases, and the immune system becomes more efficient at identifying and destroying cancer cells. Fasting may enhance the activity of natural killer (NK) cells, which play a key role in immune surveillance and tumor destruction. This immune-modulatory effect provides an additional mechanism through which fasting aids in cancer treatment, by both directly inhibiting tumor growth and improving the body's natural defense system against cancer [11].

Stress resistance in normal cells

While fasting increases the vulnerability of cancer cells to treatment, it simultaneously creates a protective, stress-resistant state in normal cells. This dual action, making cancer cells more susceptible to damage while protecting healthy cells, has significant implications for cancer therapy. Fasting decreases the growth signals in normal cells, effectively slowing down their metabolic processes and reducing the likelihood of damage from chemotherapy and radiotherapy. This stress-resistant state may allow for higher doses or more aggressive treatment regimens without increasing toxicity to the patient.

Research studies have shown that normal cells can enter a phase of maintenance during fasting time, where they conserve energy and resources, allowing them to recover more easily from treatment-induced stress [12]. Moreover, the cancerous cells, which are unable to slow down their growth pattern, continue to divide and divide and thus become more exposed to the cytotoxic effects of treatments. This differential response between normal cells and cancerous cells is one of the most promising aspects of integrating fasting into cancer therapy, as it could lead to more effective treatments with fewer side effects [13]. The mechanistic insights that are followed in the fasting regime as a treatment for cancer reveal a complex interplay between metabolism, cellular stress responses, and immune function. Fasting induces autophagy by enhancing the immune activity and creating a metabolic environment, inhibiting the growth of cancer cells while protecting normal cells. These processes make fasting a very compelling complementary therapy in oncology, particularly in combination with chemotherapy and radiotherapy [14]. However, while many preclinical studies and clinical trials suggest promising outcomes of this fasting, further research is needed to fully understand the long-term mechanism and safety of fasting as part of a cancer treatment regimen. Fasting may not be the only solution, but its ability to exploit the metabolic vulnerabilities of cancer cells while safeguarding normal cells presents a unique opportunity to improve cancer treatment outcomes.

Evidence from animal models and in vitro studies

Fasting has been extensively studied in cancer, particularly in animal models, to understand its exact potential effects on cancer progression and in its treatment. These studies have shown promising results, indicating that fasting could reduce tumor growth cells, by enhancing the sensitivity of cancer cells to chemotherapy and radiation, and even improve the survival rates in rodents. The compelling evidence that came out from these studies has laid the foundation for exploring fasting as an adjunctive strategy in cancer therapy, although translating these findings to human clinical practice remains a challenge [15,16].

Tumor growth reduction

Fasting has been shown to play a significant role in reducing tumor growth, primarily through metabolic adaptations that deprive cancer cells of the nutrients required for their rapid proliferation. Animal model studies have demonstrated that fasting triggers systemic changes, including reduced glucose availability and altered insulin and IGF-1 signaling, which can restrict the energy supply to tumor cells, thereby inhibiting their progression [16]. A notable study in mice with various tumor types found that periodic fasting cycles significantly slowed tumor growth rates. This effect was particularly pronounced in highly glycolytic cancers, such as breast and lung tumors, which rely heavily on glucose metabolism for survival [16]. By lowering circulating glucose and insulin levels, fasting disrupts the metabolic pathways essential for these tumors, thereby suppressing their growth. Additionally, fasting has been found to enhance the effectiveness of conventional cancer therapies. The same study observed that, when fasting was combined with chemotherapy, the reduction in tumor size was significantly greater compared to chemotherapy alone [17]. This suggests that fasting creates a metabolic environment that not only inhibits tumor progression but also increases cancer cell sensitivity to therapeutic interventions. One proposed mechanism is that fasting promotes differential stress resistance, where healthy cells enter a protective state under nutrient deprivation, while cancer cells, which lack this adaptive ability, become more vulnerable to treatment-induced damage. These findings highlight the potential of fasting as a complementary strategy in oncology, offering a non-invasive approach to slowing tumor growth and enhancing treatment efficacy. Clinical research is needed in the future to determine optimal fasting protocols and to explore their integration into standard cancer treatment regimens [17].

Fasting-induced synergistic effects on cancer therapies

Preclinical studies have shown that fasting elevates the sensitivity of cancer cells to chemotherapy and radiation therapy, making these treatments more effective [18]. Chemotherapy and radiation work by causing damage to rapidly dividing cells, which include both cancer cells and, to a lesser extent, normal cells. Moreover, fasting appears to differentially protect normal cells while making cancer cells more vulnerable to these therapies. The underlying mechanism behind this enhanced sensitivity is linked to metabolic stress; that is, fasting imposes on cancer cells. Further, the cancer cells, already under metabolic strain due to their rapid growth, struggle to cope with the additional stress of nutrient deprivation caused by fasting. As a result, they become more susceptible to the DNA damage caused by chemotherapy and radiation therapy [19]. In contrast, normal cells begin to enter a stress-resistant state by fasting, slowing down their metabolic activity, and making them less vulnerable to treatment-induced damage. This phenomenon is termed differential stress resistance and stands as a key advantage of fasting in cancer therapy, as it allows for more aggressive treatment without elevating the toxicity to healthy cells. In a notable rodent study, researchers found that short-term fasting before chemotherapy led to a dramatic improvement in treatment efficacy [19]. Mice that underwent fasting before receiving chemotherapy experienced greater tumor shrinkage and prolonged survival compared to those that were fed normally. Importantly, fasting also reduces the toxic side effects of chemotherapy, such as weight loss and damage to the gastrointestinal tract, which are common concerns in cancer treatment. These findings highlight the potential of fasting to not only enhance the effectiveness of chemotherapy but also improve the overall well-being of patients during treatment [20].

Cellular recycling and immune system regulation in cancer

Fasting-induced autophagy, the process by which cells break down and recycle damaged components, is another critical mechanism that contributes to its anti-cancer effects. Autophagy is upregulated during fasting, allowing the body to clear out dysfunctional or cancerous cells while promoting the regeneration of healthier ones. In animal models, fasting has been shown to increase autophagic activity in tumors, leading to a reduction in tumor size and a delay in cancer progression [21]. In addition to promoting autophagy, fasting appears to modulate the immune system, enhancing the body's natural ability to fight cancer. Studies in rodents have demonstrated that fasting can boost the activity of immune cells, particularly NK cells and cytotoxic T cells, which are responsible for identifying and destroying cancer cells [21]. In one study, fasting was found to increase the infiltration of NK cells into tumors, leading to a more robust immune response against cancer. This immune modulation could provide an additional layer of protection against cancer, complementing the direct anti-tumor effects of fasting [22].

Fasting-mimicking diets (FMDs) and caloric restriction

Beyond complete fasting, FMDs and caloric restriction have also been explored in preclinical studies as less extreme but potentially effective alternatives. FMDs are designed to replicate the metabolic effects of fasting without requiring total abstinence from food. In rodent models, FMDs have shown similar benefits to fasting, including reduced tumor growth, increased chemotherapy efficacy, and improved survival rates. These diets may offer a more practical approach to incorporating fasting-like interventions into cancer treatment, particularly for patients who may not tolerate prolonged fasting [23].

Examples of FMDs

ProLon diet: A commercially available five-day plant-based diet developed by Dr. Valter Longo. It provides around 750-1100 kcal per day, with a composition rich in healthy fats, moderate carbohydrates, and minimal protein to mimic fasting effects.

Ketogenic diet: A high-fat, low-carbohydrate diet that induces a fasting-like metabolic state by promoting ketone body production, leading to cellular stress resistance and potential anti-cancer effects.

These diets offer a more feasible alternative to prolonged fasting while retaining many of its therapeutic benefits, including improved metabolic health and enhanced response to cancer therapies.

Caloric restriction, which involves reducing overall calorie intake without fasting completely, has also been studied in animal models of cancer. Like fasting, caloric restriction has been shown to inhibit cancer cell growth and enhance treatment efficacy. However, the degree of benefit appears to vary depending on the type of cancer and the specific caloric restriction protocol used. Preclinical studies in animal models and in vitro experiments provide strong evidence that fasting can reduce tumor growth, increase the sensitivity of cancer cells to chemotherapy and radiation, and improve survival rates [23]. The metabolic stress imposed by fasting deprives cancer cells of essential nutrients, while normal cells enter a protective state, reducing the side effects of treatment. Fasting’s anti-cancer potential is further strengthened by its role in cellular recycling and immune system regulation. While current findings are encouraging, further research is essential to translate these benefits into clinical oncology. More human trials are necessary to evaluate the safety, effectiveness, and practical application of fasting and FMDs across various cancer types and patient populations. Despite these challenges, preclinical studies indicate that fasting could serve as a valuable adjunct to conventional cancer treatments [24].

Short-term fasting and chemotherapy tolerance

A number of small-scale clinical studies have explored the impact of short-term fasting (typically 24-72 hours) on cancer patients undergoing chemotherapy. These studies have sought to determine whether fasting can reduce the toxicity associated with chemotherapy, such as fatigue, nausea, and gastrointestinal discomfort, while improving the body’s response to treatment. One of the key findings from these trials is that short-term fasting may indeed improve patients' tolerance to chemotherapy. In a study involving breast cancer patients, those who fasted for 48 hours before and after chemotherapy reported fewer side effects compared to those who followed a normal diet. Patients who fasted experienced less nausea, fatigue, and weakness, common side effects of chemotherapy [25]. Moreover, some studies suggest that fasting may protect healthy cells from the damaging effects of chemotherapy, a phenomenon known as "differential stress resistance." Fasting induces a metabolic shift in normal cells that allows them to enter a stress-resistant state, reducing the collateral damage caused by chemotherapy while leaving cancer cells more vulnerable [26]. In a separate trial involving patients with a variety of cancer types, fasting for up to 72 hours was associated with a reduction in chemotherapy-induced side effects, such as mucositis (inflammation of the digestive tract lining) and hematologic toxicity (damage to blood cells). While these findings are encouraging, they are based on relatively small sample sizes and require further validation through larger, more rigorous studies [27].

Nutritional strategies imitating fasting: a practical approach

FMDs are designed to replicate the metabolic effects of fasting without requiring patients to completely abstain from food. These diets typically involve low-calorie, low-protein, high-fat meals that simulate a fasting state by reducing glucose and insulin-like IGF-1 levels. FMDs have been proposed as a more practical alternative to complete fasting, particularly for cancer patients who may be unable to tolerate prolonged periods without food due to malnutrition or other health concerns [28]. Several early-phase clinical trials have investigated the potential benefits of FMDs in cancer therapy. In a pilot study conducted on patients with breast cancer, those who followed a five-day FMD during chemotherapy cycles showed improved tolerance to treatment, reporting fewer side effects, such as fatigue, nausea, and dizziness. Moreover, markers of cellular stress, such as IGF-1, were reduced in the FMD group, suggesting that the diet may create a less favorable environment for cancer cell proliferation. While these results are promising, the study was small, and additional research is needed to confirm these findings in larger patient populations [28]. Although some clinical trials have demonstrated that fasting can improve patients' quality of life during cancer treatment, its direct impact on tumor reduction and survival rates remains uncertain. The majority of studies have focused on fasting's ability to mitigate side effects and improve treatment tolerability rather than on fasting as a standalone intervention to shrink tumors or improve overall survival outcomes. In one of the few trials examining the effects of fasting on cancer outcomes, patients with advanced cancer who fasted for 24 hours before and after chemotherapy did not experience significant tumor shrinkage compared to those who did not fast [20]. However, fasting appeared to have a positive effect on patients' overall well-being, with many reporting improved energy levels and reduced treatment-related fatigue. Importantly, fasting did not appear to compromise the effectiveness of chemotherapy, suggesting that it may be a safe adjunct to conventional treatment regimens. However, larger, long-term studies are needed to determine whether fasting can directly influence cancer progression and survival rates in humans [20].

Obstacles and considerations in clinical research

Several challenges have limited the widespread adoption of fasting in clinical oncology. One of the primary concerns is the potential for fasting to exacerbate malnutrition in cancer patients, many of whom are already at risk of weight loss and muscle wasting due to their disease. Prolonged fasting could lead to further nutritional deficits, particularly in patients with advanced cancer, who may have limited reserves of energy. To mitigate these risks, most clinical trials have focused on short-term fasting or FMDs, which are less likely to cause severe malnutrition [23]. Another limitation is the small sample size of many human trials on fasting and cancer. Most studies to date have involved fewer than 100 participants, making it difficult to draw definitive conclusions about the safety and efficacy of fasting in larger, more diverse patient populations. Furthermore, the heterogeneity of cancer types and treatment regimens in these trials makes it challenging to generalize the findings to all cancer patients. Future studies will need to address these limitations by recruiting larger cohorts and exploring the effects of fasting in specific cancer subtypes, such as breast, lung, or colorectal cancer. While small-scale clinical studies suggest that short-term fasting or FMDs may help cancer patients tolerate chemotherapy better and experience fewer treatment-related side effects, the evidence supporting fasting’s direct impact on cancer outcomes, such as tumor reduction and survival rates, remains limited [23]. Current clinical trials have primarily focused on fasting as an adjunct therapy rather than a primary treatment, and more research is needed to establish its safety and efficacy in larger, more rigorous studies [29]. For now, fasting shows promise as a complementary approach in cancer care, but it is not yet robust enough to be considered a standalone treatment.

A supportive therapeutic approach

Fasting has emerged as a potential complementary therapy in the treatment of cancer, offering a way to enhance the efficacy of traditional treatments, such as chemotherapy and radiation. While fasting is not a standalone cure for cancer, research suggests that it may help mitigate some of the adverse side effects associated with conventional cancer therapies, making them more tolerable for patients. This, in turn, could lead to better overall outcomes by allowing for higher doses or more frequent treatment cycles without compromising patient health. As such, fasting is increasingly being explored as an adjunctive measure to improve patient quality of life and potentially enhance treatment efficacy [30].

Minimizing therapy-induced adverse effects

Chemotherapy and radiation are effective cancer treatments but come with significant side effects, including nausea, fatigue, and gastrointestinal issues, which can diminish patients’ quality of life and limit the intensity or frequency of treatment. Some studies have shown that fasting or FMDs can alleviate these side effects, offering a reprieve for patients undergoing aggressive treatment. The mechanism behind this benefit is thought to be linked to the body’s response to nutrient deprivation [30]. Fasting triggers a protective, stress-resistant state in normal cells, which makes them less vulnerable to the damaging effects of chemotherapy and radiation. This phenomenon, known as “differential stress resistance,” may allow normal cells to better withstand treatment while leaving cancer cells more susceptible to damage. For instance, in several small-scale human trials, patients who fasted before and after chemotherapy reported fewer side effects such as fatigue, nausea, and diarrhea. By reducing these debilitating side effects, fasting could help patients tolerate more intensive treatment schedules, which might improve cancer outcomes by allowing for more aggressive treatment strategies [30].

Boosting cancer treatment outcomes with metabolic interventions

Fasting may not only reduce the side effects of chemotherapy and radiation but also improve their effectiveness. Cancer cells are known for their rapid growth and high metabolic demand, particularly their reliance on glucose as an energy source (a process known as the Warburg effect). Fasting deprives cancer cells of glucose, creating a metabolic environment that is unfavorable for their survival. While normal cells can switch to alternative energy sources during fasting, cancer cells are less adaptable, making them more vulnerable to the effects of nutrient deprivation [31]. This metabolic stress could enhance the ability of chemotherapy and radiation to kill cancer cells more effectively. Moreover, some studies suggest that fasting may increase the sensitivity of cancer cells to treatment. For instance, in rodent models, fasting has been shown to boost the efficacy of chemotherapy by making cancer cells more vulnerable to the DNA damage induced by treatment [31]. Although more clinical research is needed to confirm these findings in humans, the preliminary data are promising, particularly for cancers that are highly dependent on glucose metabolism.

Integrating fasting into cancer care: precautions and clinical guidance

While fasting shows promise as a complementary therapy, it is not without risks. Cancer patients are often in a weakened state and may already be experiencing malnutrition due to their illness and treatment. Prolonged fasting could exacerbate these conditions, leading to weight loss, muscle wasting, and a weakened immune system [32]. As such, fasting should always be done under medical supervision, especially for cancer patients. These diets have shown similar benefits in reducing chemotherapy side effects and enhancing treatment efficacy while being easier to implement, particularly for patients who cannot tolerate long periods without food [32]. While fasting should not be viewed as a cure for cancer, it may help patients better tolerate conventional treatments, thereby improving overall outcomes. However, fasting, particularly in cancer patients, should be approached cautiously and under the guidance of medical professionals to avoid exacerbating malnutrition or other health risks [33]. As research into fasting and FMDs continues, it may become an important tool in the oncology toolkit, improving both patient experience and treatment success [33].

Apoptosis and its role in fasting-induced cancer modulation

Apoptosis, a highly regulated form of programmed cell death, plays a crucial role in maintaining cellular homeostasis and eliminating damaged or cancerous cells. Emerging evidence suggests that fasting may enhance apoptotic pathways, thereby contributing to cancer suppression. Several studies indicate that fasting modulates key apoptotic signaling pathways, including the intrinsic (mitochondrial) and extrinsic (death receptor-mediated) pathways, ultimately leading to tumor cell elimination [34]. Additionally, fasting-induced metabolic stress triggers pro-apoptotic proteins such as p53, BAX, and caspase activation while downregulating anti-apoptotic factors such as Bcl-2, thereby sensitizing cancer cells to apoptosis. This mechanism is further supported by a recent review highlighting fasting as a potential adjunct therapy in oncology, where apoptosis is a key driver of its therapeutic benefits [32]. Given these findings, a deeper exploration of apoptosis within the context of fasting would provide a more comprehensive understanding of its implications in cancer treatment, particularly in combination with conventional therapies [32].

## Conclusions

In conclusion, fasting as a cancer treatment holds significant potential, particularly as an adjunctive therapy to enhance the efficacy of traditional cancer treatments. The mechanisms by which fasting may impact cancer progression - such as autophagy, metabolic regulation, and stress resistance - are compelling. However, the lack of large-scale human trials and the potential risks of malnutrition make fasting a complementary approach rather than a primary treatment strategy. Further research is needed to fully understand the therapeutic potential of fasting in oncology and to identify the patient populations and cancer types that might benefit the most. Until more conclusive evidence emerges, fasting should be approached cautiously, with careful oversight by healthcare professionals to ensure patient safety and avoid adverse effects.
